# Transplacental Antibody Transfer of Respiratory Syncytial Virus Specific IgG in Non-Human Primate Mother-Infant Pairs

**DOI:** 10.3390/pathogens10111441

**Published:** 2021-11-05

**Authors:** Michael P. Citron, Jessica McAnulty, Cheryl Callahan, Walter Knapp, Jane Fontenot, Pablo Morales, Jessica A. Flynn, Cameron M. Douglas, Amy S. Espeseth

**Affiliations:** 1 Infectious Disease & Vaccines, Merck & Co., Inc., Kenilworth, NJ 07033, USA; jlmc@umich.edu (J.M.); Cheryl_Callahan@merck.com (C.C.); jessica_flynn@merck.com (J.A.F.); cbsdouglas@comcast.net (C.M.D.); amy_espeseth@merck.com (A.S.E.); 2 Safety Assessment and Laboratory Animal Resources, Merck & Co., Inc., Kenilworth, NJ 07033, USA; walter_knapp@merck.com; 3 The New Iberia Research Center, University of Louisiana at Lafayette, New Iberia, LA 70560, USA; jfontenot@louisiana.edu; 4 The Mannheimer Foundation, Homestead, FL 33034, USA; pmorales@mannheimerfoundation.org

**Keywords:** maternal antibody, animal model, respiratory syncytial virus (RSV), non-human primate, maternal immunization

## Abstract

One approach to protect new-borns against respiratory syncytial virus (RSV) is to vaccinate pregnant women in the last trimester of pregnancy. The boosting of circulating antibodies which can be transferred to the foetus would offer immune protection against the virus and ultimately the disease. Since non-human primates (NHPs) have similar reproductive anatomy, physiology, and antibody architecture and kinetics to humans, we utilized this preclinical species to evaluate maternal immunization (MI) using an RSV F subunit vaccine. Three species of NHPs known for their ability to be infected with human RSV in experimental challenge studies were tested for RSV-specific antibodies. African green monkeys had the highest overall antibody levels of the old-world monkeys evaluated and they gave birth to offspring with anti-RSV titers that were proportional to their mother. These higher overall antibody levels are associated with greater durability found in their offspring. Immunization of RSV seropositive AGMs during late pregnancy boosts RSV titers, which consequentially results in significantly higher titers in the vaccinated new-borns compared to the new-borns of unvaccinated mothers. These findings, accomplished in small treatment group sizes, demonstrate a model that provides an efficient, resource sparing and translatable preclinical in vivo system for evaluating vaccine candidates for maternal immunization.

## 1. Introduction

Human RSV is a leading cause of bronchiolitis and pneumonia in infants and acute respiratory disease in infants and young children [1,2]. Each year, RSV results in an estimated 34 million cases of acute lower respiratory infection (ALRI), 3.4 million hospitalizations and almost 60,000 deaths worldwide [3,4]. Despite the high incidence rate and burden on both the health system and patients, no licensed vaccine is available. Antiviral ribavirin [5] and other palliative measures, such as oxygen therapy and the now discontinued RSV specific immune globulin, RSV-IVIG product (RespiGam®, MedImmune Inc., Gaithersburg, MD, USA), have been used for severe cases of disease, and a monoclonal antibody, Palivizumab, has been used for prophylaxis in high-risk infants. There are several obstacles facing the development of an RSV vaccine, with no licensed vaccine against RSV despite six decades of effort [6]. In the youngest population, maternally derived anti-RSV antibodies have been shown to have an inhibitory effect on immunization with RSV vaccines [7,8,9]. Additionally, there are lingering safety concerns associated with the vaccination of RSV-naïve infants and toddlers, particularly regarding subunit modalities. These concerns arose due to a formalin-inactivated RSV (FI-RSV) vaccine, which, when administered to seronegative children, resulted in vaccine-enhanced respiratory disease following natural infection [10,11]. Therefore, other attractive strategies have emerged to overcome these obstacles, including the immunization of pregnant women intended to boost functional antibody at the latter stages of pregnancy. Although the protection would be temporary due to the kinetics of the transferred antibodies [12,13,14], the benefits would be large since the first six months of pregnancy are the most vulnerable, and at this time the incidence of bronchiolitis is at its highest, as it coincides with the small size of the new-born’s airways. A relevant preclinical animal model that can mimic humans and predict clinical outcomes could provide a desired tool for evaluating the promise of MI for a vaccine against targets, such as RSV. Because of their relatedness to humans and studies showing the promise that monkeys could provide for other vaccine candidates, we proposed old-world monkeys would be a valuable nonclinical resource for the devolvement of a vaccine candidate against RSV. Our study demonstrates that the African Green Monkey is uniquely capable of providing an efficient transfer of maternal antibodies and can be a power model to test protection against viral infection in studies designed for small group sizes, which are often considered a barrier for large animal efficacy models.

Maternally derived RSV antibodies are efficiently transferred to the foetus and have been shown to be inversely associated with acute lower respiratory tract RSV infection and disease severity [15,16]. Ensuring an abundance of high quantity, neutralizing antibodies through the first several months of life could substantially lessen the disease burden in new-borns [17]. Maternal immunization (MI), or immunization during pregnancy has been a common practice resulting in substantial global impact by providing protection against infectious disease for the mother, child, or both [18,19,20,21,22,23]. ACIP recommendations for vaccination during pregnancy against influenza, tetanus, and pertussis, provide a pathway allowing a safe and generally well tolerated MI strategy against RSV to be feasible [24,25,26,27,28]. The result would allow us to overcome obstacles that exist for paediatric vaccination against RSV, and greatly benefit the expecting mother [29,30].

Several vaccines against RSV using the MI approach have been [31] or are currently under evaluation clinically, including GlaxoSmithKline (NCT02753413), Pfizer (NCT03529773), and the NIAID (NCT03049488). Since history suggests that the path to licensure against this disease is uncertain, there continues to be a need for translatable tools to assess candidate vaccines. Animal models, where maternal antibody transfer occurs with similar timing and efficiency to humans, would be an important example. Recently, mouse and cotton rat models of RSV infection have been utilized to test maternal immunization using RSV vaccines [32,33,34]. However, rodents and even some larger animals, have inherent disadvantages for evaluating MI. Mice and humans have distinct foetal physiology and placentation. Mice have a short gestation period, a different mechanism of implantation, a prominent yok sac, fewer placental hormones, and shallow trophoblast invasion, and maternally derived IgG is transferred to the mouse postnatally [35]. Furthermore, humans and rodents differ in FcRN expression profiles, antibody binding affinity, and susceptibility to infection [36,37]. Non-human primates (NHP), in contrast, might possess the advantage of being able to mimic the unusual interstitial implantation of human and placenta architecture. Macaques and baboons have similar structural homologies, such as the trophoblast and spiral arteries interaction [38].

Here, we demonstrate the positive attributes that support the use of the NHP as a strong model for MI. First, NHPs can seroconvert following both experimental and natural infection with human RSV. Second, new-born monkeys have similar antibody titers to the corresponding dam at the time of birth. These pre-existing antibodies can be boosted during pregnancy with an adjuvanted RSV subunit vaccine and the subsequent offspring have higher anti-RSV IgG antibody titers at birth compared to offspring from unvaccinated mothers. Importantly, low numbers of animals appear to be needed due to the low variability and large difference between animals and between groups, respectively. The data from these non-terminal, reusable resources suggest NAb can cross the placenta in NHPs by passive transfer, thus providing a translatable system to evaluate the utility of candidate vaccines for the MI route of administration. 

## 2. Results

### 2.1. Papio Hamadryas Baboons Lack Robust Antibody Levels against Natural RSV Infection, Independent of Age

**Figure 1:**
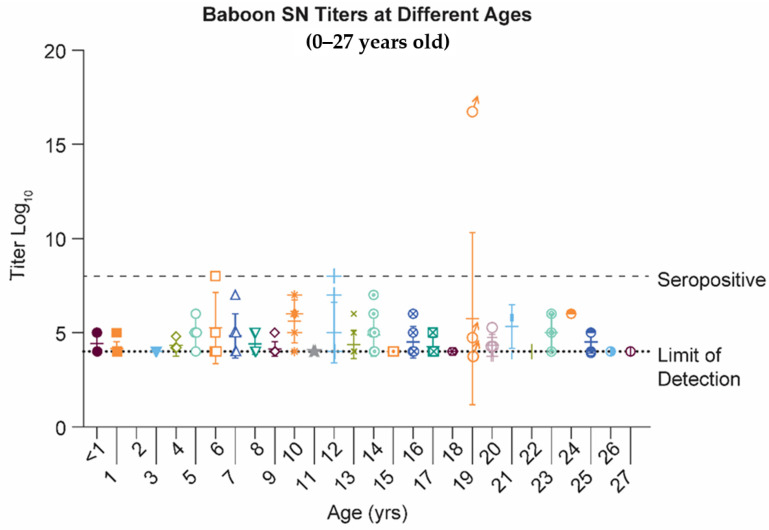
Serum neutralization titers of 27 baboons (hamadryas) were collected and examined for neutralizing antibody titers against RSV. Although baboons have been reported as being able to be infected with RSV, this colony of baboons was not observed to have appreciable antibody titers above background. Seropositive level set as 2-fold over background.

Because baboons have been shown to mimic the transfer of protective maternal antibodies in neonates [39,40,41,42], we chose to evaluate the feasibility to establish an MI model for RSV vaccines in the baboon. Twenty-seven baboons, ranging between new-borns to 27 years of age were serologically tested for neutralizing antibodies against the RSV. Only three were at or above the seropositive cut-off of 8, or 2-fold over the limit of detection (Figure 1). The three animals were all adults: 6, 12 and 19 years of age.

### 2.2. African Green Monkeys Demonstrate Antibody Levels against Natural and Experimental RSV Infection That Make Them Suitable to Evaluate Maternal Immunization for RSV Vaccines

**Figure 2:**
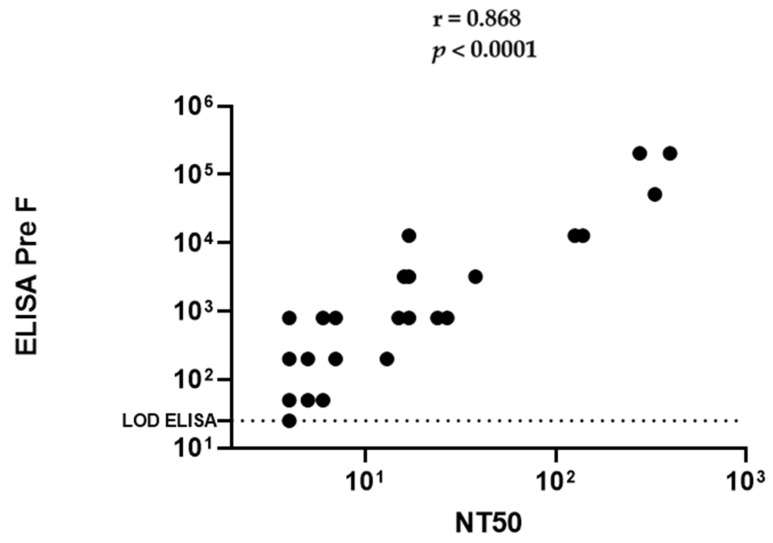
Correlation analysis of specific binding antibody responses versus neutralization titers. Correlation analysis shows serum neutralizing antibody (NT50) titers (x-axis) versus PreF-specific IgG titers on y-axis for 50 AGM individual animals. Dotted line on x-axis and y-axis indicates limit of detection. Correlations were calculated by Spearman’s correlation coefficient. R = 0.7381 (0.6178 to 0.8643 95% confidence interval); r. *p* ≤ 0.0001 was considered significant. Some values overlap and do not show up an individual data point, but data considers 50 animals.

**Figure 3:**
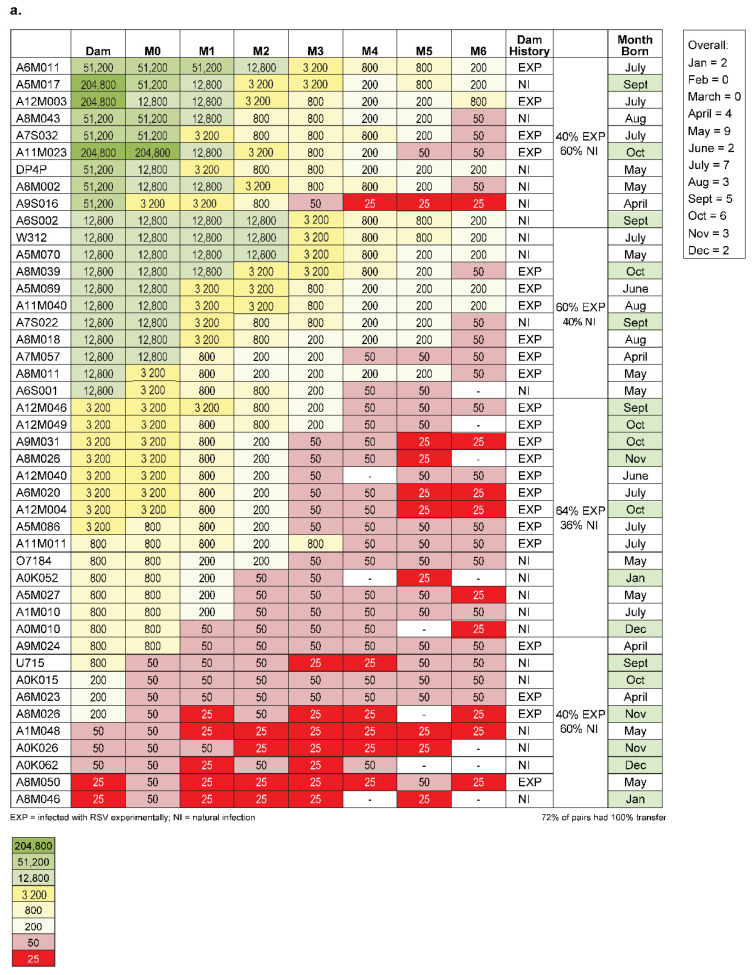

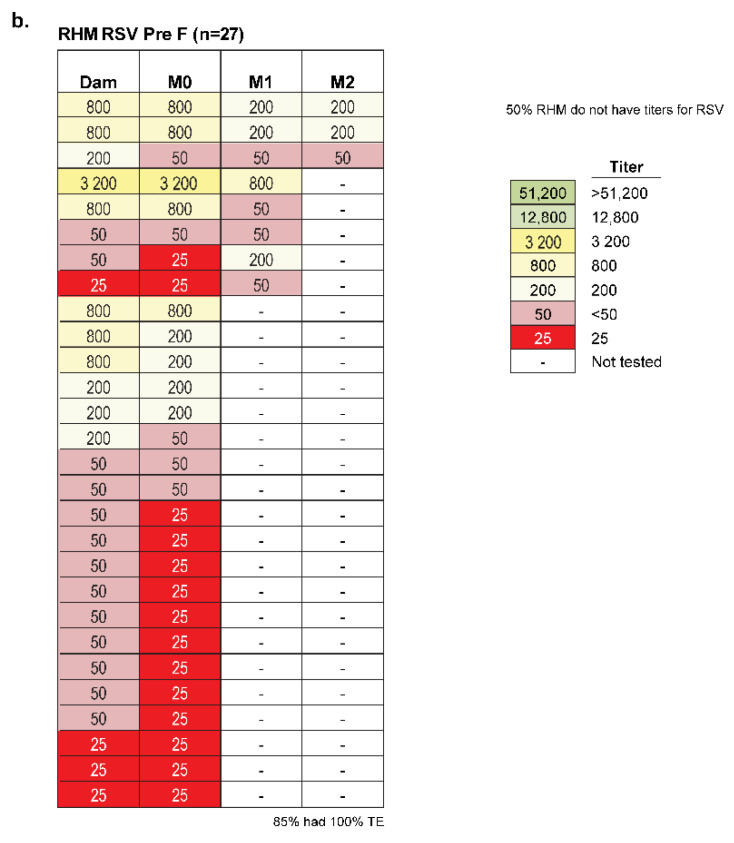
IgG end point binding titers (anti-PreF) shown for (**a**) Forty-four randomly assigned African Green Monkeys (AGM) and (**b**) twenty-eight randomly assigned Rhesus macaques (RHM) with each corresponding offspring. The infant was tested for anti-PreF titers at birth and each month following. Titers are listed for animal that were either experimentally inoculated with RSV A2 virus or naturally seroconverted. M0 = birth, M1 = one month old, M2 = two months old, M3 = three months old, M4 = four months old, M5 = five months old and M6 = six months old; TE = transfer efficiency.

Since these baboons, as a group, unexpectedly demonstrated low antibody responses, we elected to test monkey species that have previously shown immunologically responses to RSV [43,44]. We do not understand if *Papio hamadryas*, used here are less susceptible to human RSV (hRSV) or if the titers observed were a result of the husbandry practices in the closed colony used. Interestingly, we observed less robust binding and Nab levels and virus in the nose and lungs in Rhesus compared to AGM following challenge with hRSV, and do not know if this outcome is similar to what we speculated for the baboon. We tested if monkeys naturally seroconvert and can passively transfer antibodies to their new-born. We quantified the levels of RSV specific antibody in a panel of twenty-eight Rhesus and fifty African Green randomly selected and presumed pregnant. Once born, the mother and baby were bled for serum starting on the day of birth and then monthly thereafter for twelve months. We demonstrated that binding IgG titers via a solid phase immunoassay ELISA correlate well with the cell-based serum neutralization assay (Figure 2). Therefore, we decided to evaluate all the samples with ELISA. Figure 3a,b show that monkeys not experimentally infected with RSV generate anti-RSV antibodies naturally. The maximum antibody titer for AGM was 1:204,800 and 1:3200 for Rhesus. The AGMs were about sixty-four-fold higher in antibody level compared to Rhesus. Moreover, the higher titers observed in African greens are associated with similar antibody levels in their respective offspring, detectable at least 4 to 5 months following birth (Figure 3a). In contrast, the lower overall magnitude of titers observed in the Rhesus infants are barely detectable after one month of life. Binding titers below 10^3^ are correlated with lower or undetectable neutralizing antibodies, therefore resulting in the expectation of being vulnerable to infection as well as disease.

### 2.3. Amplitude of Infant Serostatus Is Independent of the Manner of How Animals Seroconverted

In addition, we evaluated if antibody levels observed in the infant had differences associated with how the mother’s immune status was acquired. We measured animals which were either experimentally infected with RSV or had pre-existing anti-RSV antibody titers, presumably because of natural infection. Figure 3a shows that there is a lack of relationship between the method of seroconversion and the level of antibody titer since there is no apparent association between the level of antibody in the offspring and how seroconversion resulted. New-borns had titers that were equivalent or within the assay limit of 4-fold to their respective mothers, independent of seroconversion method. Forty percent of animals with the highest quartile of binding titers were seropositive due to experimental infection with RSV A2 from a previous use, while sixty percent were seropositive assumingly from natural infection since they were experimentally naïve. For the next quartile of highest titers, the split was 60% experimental (Exp) and 40% natural infection (NI). The next lowest quartile was 64% Exp and 36% NI. The lowest quartile group was 40% Exp and 60% NI.

### 2.4. No Relationship Exists between the RSV Serostatus of New-Born Monkeys and Seasonality of Birth Date

**Figure 4:**
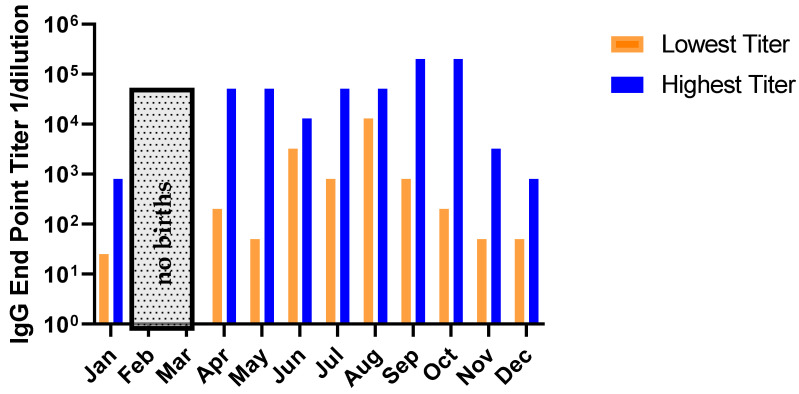
New-borns were bled at the time of birth. For each month of the year the animals that showed the highest and lowest titer were identified and represented in Figure 4. There were no births during the months of February and March. Although the animals were born in a temperate climate in the northern hemisphere, there is no apparent relationship with increased titers from new-borns and the typical peak season of RSV for the region the animals were born. This section may be divided by subheadings. It should provide a concise and precise description of the experimental results, their interpretation, as well as the experimental conclusions that can be drawn.

In order to exclude a causal relationship between infants becoming seroconverted after birth, we assessed (Figure 4) if seasonality of circulating RSV correlated with a higher rate of seroconversion in the new-borns. We found no association between the offspring titer and the month in which they were born. In North America, typically, the end of December until late January are the peak of the RSV season which, in typical years, starts in October and ends in mid-April [45,46]. The animals were born and housed in Louisiana which is known for its semi-tropical climate. Most animals positive for antibodies were born in July. There was no relationship between a high titer at birth and being born within the historical season for RSV circulation; Fall = 22 September to 21 December (*n* = 10), Winter = 22 September to 21 December to 21 March (*n* = 0), Spring = 22 March to 21 June (*n* = 12), Summer 22 June to 21 September (*n* = 14).

### 2.5. Maternal Immunization of Pregnant Monkeys Results in Offspring with Significant Increase of Level and Durability of Anti = RSV Antibody

Since AGMs can seroconvert when infected with human RSV, either intentionally via the experimental delivery of the virus or through exposure to RSV-infected handlers or other infected animals, and since those antibody titers were efficiently transferred to the neonate, it is reasonable to hypothesize that immunization of pregnant monkeys would result in increased average titers of the offspring. In human clinical trials, mothers in their third trimester were immunized with RSV F proteins with or without adjuvant [47]. Therefore, we chose to immunize pregnant AGM with RSV Prefusion F protein with Adjuphos^®^ Adjuvant (InvivoGen, San Diego, CA, USA). Pre-immunization and at the time of birth, titers were measured in the dams, along with the neonates. The infants were serologically followed monthly (Table 1). Three of the four immunized mothers gave birth two weeks or more following immunization. The three AGMs that were born at least two weeks post immunization had a geometric mean titer (GMT) of 4.55Log_10_ at the time of birth, while the antibody responses of the three mothers was 5.01Log_10_ (GMT). The increased titers from RSV vaccinated mothers versus unvaccinated animals at day 28 are significant (*p*-value < 0.001). Additionally, comparing infants born from vaccinated and unvaccinated mothers at the time of birth and 28 days later were also significantly different (*p*-value < 0.001 and 0.001, respectively). One animal gave birth the following day and lacked the higher antibody level the other three animals that were born at least two weeks post immunization. The lack of transfer of antibody from the mother is seemingly related to the short time frame between vaccination and the birth date. The unvaccinated group had the same overall titers (GMT = 221) for both the mothers and new-borns, indicating efficient transfer of pre-existing antibodies, even when titers were overall low. The high transplacental antibody transfer efficiency in mother–infant pairs in monkeys appears to be similar to humans [48,49]. Additionally, significant differences between the infants of vaccinated and unvaccinated mothers were observed despite the small numbers of animals used between and within groups. The parameters were logarithmic scale (base 10) transformed prior to analysis to reduce skewness. An analysis of variance (ANOVA) factoring for the effects of vaccinated condition (RSV-vaccinated versus unvaccinated) to assess the potential effects of RSV maternal immunization was performed. These analyses were performed in R version 3.6.0 (R Foundation for Statistical Computing, Vienna, Austria).

## 3. Discussion

While nonclinical models evaluating maternal immunization for RSV do exist, there is an opportunity for improved and highly translatable animal models for this strategy. NHPs are generally viewed as one of the most translatable laboratory animals used in vaccine discovery, research, and development; yet, they are frequently judged for specific their logistical disadvantages, such as cost. Here, we show that old-world monkey new-borns can present efficient passive transfer of boosted antibodies from mothers, using small numbers of animals to observe significant serological differences between treated and untreated groups. Furthermore, studies investigating MI strategies using NHPs for RSV and perhaps other vaccine targets, are likely to provide more translatable data due to the similar features in antibody architecture, kinetics of the antibody level and placentation that exist between human and NHP [50]. A robust preclinical tool would offer both economic incentive by developing the most promising candidates, as well as prudent use of animals in a non-evasive approach.

Immunization of women during pregnancy is one strategy to overcome many of the obstacles that linger for achieving a licensed vaccine against RSV in the young. There are several benefits for maternal immunization. First, inhibition of activities vaccination of new-borns could be avoided. Secondly, increased protection in the mother from boosted anti-RSV IgG could lead to a reduction in vertical transmission [51,52,53,54,55,56,57,58]. Third, boosted systemic immunoglobins, such as protective IgG antibodies, could be transferred to the foetus via the placenta, as well as mucosal IgG, IgA and IgM during breastfeeding [59,60,61,62]. Moreover, the theoretical risk of specific vaccine-associated enhanced respiratory disease (VAERD) could be circumvented. Lastly, a direct boost to the immunosuppressed pregnant mother could provide a benefit to the mother, who is immunologically at-risk during this time [63].

There has been notable clinical success with MI. The Advisory Committee on Immunization Practices (ACIP) recommends immunization with pertussis, influenza, and tetanus vaccines during pregnancy [64,65,66,67,68,69]. Other vaccines are recommended for emergency situations, such as outbreaks during pregnancy, including vaccines against Cholera, Rabies and Yellow Fever [70,71]. Moreover, there is a robust pipeline supporting this strategy for protecting offspring, including targets such as Group B *Streptococcus* (GBS), SARS-CoV-2 and RSV [72,73,74,75,76,77,78]. For RSV, adjuvanted, subunit post fusion (F) vaccines have been shown to be well tolerated and possess high efficiency of transplacental transfer, especially the closer vaccination was to birth, and either provided limited increases in NAb in the new-borns or increases of titer over baseline [79]. However, to date none have resulted in preventing RSV-related disease in the lower respiratory tract. Newer antigens, such as the stabilized prefusion conformation F [80], which have shown greater neutralization activity and reduced severity of disease could offer the needed advancement for success [81]. 

Mice [82,83], cotton rats [84,85], cows [86], sheep [87], in vitro cell and tissue systems and even mathematical modelling [88] have provided insight into maternal immunization [89,90,91]. We know from clinical studies that Nab levels correlate with protection from RSV disease [92,93]. Preclinical studies in mice using a chimpanzee adenoviral vector expressing RSV F protein administered intranasally was protective against RSV challenge in offspring of immunized mothers [94]. Cotton rat studies also showed transfer or antibody and protected pups of mothers immunized with live virus during pregnancy. However, each of these systems have limitations [95]. Cotton rats have been shown to have short-lived passive protection after just 4 weeks after birth [96]. In addition, mice and cotton rats are not known to acquire antibodies from natural infection, limiting their translatability with humans. Cows [97] and mice [98,99,100] do not mediate transplacental transfer via FcRn as do humans, old-world monkeys and some other mammals. Maternofetal transfer of IgG is a complex process and is differentially regulated in each species [101]. Because of these barriers as well as the advantages that other species that may have greater theoretical translatable potential, we decided to use the NHP to evaluate the performance of vaccines for maternal immunization. NHPs have several features making them a suitable animal for evaluating MI. Baboons and macaques have a haemochorial and bidiscordial placenta, respectively. Similar to humans, these old-world monkeys display structures such as villous tree, an intervillous space, and uterine spiral arteries [102]. They also maintain a human-like FcRn. Macaques have been shown to maternally transfer IgG [103] and viruses such as Zika and cytomegalovirus [104,105,106,107]. Congenital transmission blocking of herpes viruses using MI in NHP has been demonstrated. [108,109,110]. Since several human pathogens have been able to infect NHPs [111,112] efficacy assessment of MI is feasible. For RSV, we and others have shown that monkeys seroconvert after hRSV both naturally as well as experimentally [113,114]. Therefore, until licensure of vaccine against RSV in the youngest of populations materializes, an unmet medical need remains. Nonclinical studies will continue to play a role in discovering and developing safe and efficacious vaccines. The NHP could be a highly attractive asset to facilitate the existing gap.

To understand if NHPs are a suitable species to explore maternal immunization strategies with vaccine candidates against RSV, we first evaluated baboons. Baboons, similar to humans, have four immunoglobulin G sub-classes, the ability to transfer IgG through the placenta to the foetus and a reproductive anatomy that is comparable [115,116]. However, in the colony we tested, the baboon mostly lacked (*n* = 3/27) neutralizing RSV antibody responses above background (Figure 1). Therefore, we decided to examine other old-world monkeys, the Rhesus macaque and the African Green. The Rhesus macaque (*Macaca mulatta*) and the African Green monkey (*Chlorocebus sabaeus*) have also been used in efficacy studies for anti-RSV treatments and prophylactics [117]. When we inoculated seronegative NHPs with 5.5log10 RSV A2 virus, we observed at least 5log10 plaque forming units (pfu) of virus in AGM compared to no virus in Rhesus in the bronchoalveolar lavage fluid (BAL), seven days post administration. The cause of the difference in the permissiveness is unknown. It is possible that low levels of pre-existing antibodies in Rhesus exist, which are beyond the limit of detection by our current detection methods. These results are consistent with the unremarkable detection of anti-RSV antibodies in the adult Rhesus we tested, which are relatively low compared to titers observed in AGMs. 

We tested a panel of forty-four (44) and twenty-seven (27) samples from presumed pregnant AGMs and Rhesus, respectively. The animals were randomly chosen, irrespective of their experimental history, age, or weight. All were evaluated in an anti-RSV PreF IgG ELISA. Since selective samples showed a strong correlation between serum neutralization and ELISA binding antibody titer (Figure 2) we choose to use the solid phase ELISA for all the subsequent analysis going forward. All pregnancies transpired and both mothers and infants were bled for serum. We were able to evaluate serologically the antibody level for the mother at birth as well as the new-borns. Offspring were additionally serologically evaluated each month unless the antibody levels plateaued. For AGM monkeys we observed a large range of titers in the mothers, from 5.3log10 to lower limit of detection of 25. With very few exceptions the titer of the new-born matched that of the mother, demonstrating excellent maternofoetal transfer efficiency. The Rhesus surveyed, however, had overall lower titers. Although there were few Rhesus that possessed a level of antibody that was sufficiently above background, those animals that did had comparable levels in their respective new-born, suggesting that Rhesus did passively transfer maternal antibody efficiently to their offspring (Figure 3b).

Because the animals were purposely bred animals, we have the history for each animal. We observed that about half of the animals that gave birth were naturally seroconverted, presumably from care-handlers or transmission from nearby shedding animals. The others were experimentally seropositive from RSV A2 inoculation from prior study enrolment. The magnitude of the antibody titers was independent of how seroconversion was achieved (Figure 3a). Additionally, we were able to establish that the responses observed in the infants was not due to an anamnestic response from active infection. We evaluated if monkeys born during RSV season would have higher RSV titers compared with animals born outside of the season. RSV seasonality around the globe is different depending on the geographic region of interest. In general, temperate climates have November to March in the northern hemispheres and May until July in the southern hemisphere as the peak RSV season. In the tropical regions, the duration is more evenly distributed throughout the year and quite variable [118]. In our study, animals were born throughout the year. AGMs are known to be seasonal breeders, with most births in North America in the May-July time frame, which would coincide with low RSV circulating in a typical year. Since the animals resided in a more tropical northern hemisphere region, it is noteworthy that the animals with higher titers at birth are distributed throughout the year with variability (Figure 4). We do not completely understand if animals are infected by their caretakers or how efficient RSV is spread between animals, but we surmise this process of infection occurs.

AGM had good antibody levels with efficient maternal-foetal transfer; therefore, we immunized expecting AGM mothers thought to be in the last trimester (day 111–165 days of gestation) with RSV F protein with AdjuPhos. We showed that increasing the quantity of antibody in the mother prior birth resulted in an increase for the group of new-borns in the treated group. The anti-RSV F antibody detected in mothers that gave birth at least 2 weeks following immunization had a 2.7log_10_ increase from their base line titer, as a group. Noteworthy was the fact that the new-born group had anti-RSV antibody levels about 2.2log_10_ higher than the animals born to unimmunized mothers. Interestingly, the single animal who was immunized coincidentally a day prior birth lacked similar titers observed in the other three immunized animals from the vaccinated cohort. While the mother had a boosted response, roughly 20-fold, one month from baseline, the titers from the new-born show a response similar to those born from unvaccinated mothers, suggesting that the time required for maternofoetal transfer was restricted. A follow-up study might be to challenge animals with RSV to determine the ability of these passively transferred antibodies to reduce RSV in the upper and lower respiratory tracts. It would be very interesting to know the durability of these antibodies by infecting animals at intervals of four months or greater, since humans are most vulnerable before the age of four months because of their inability to mount effective neutralizing antibody response prior. (Sande et al. 2014).

Interestingly, there is a lack of consistency between antibody half-life in monkeys and humans. It is known in NHPs that antibody levels decrease within the first three months of life and then increase gradually until about five years of age and plateau [119]. The half-life in NHPs has been estimated to be approximately 8.3 days [120] compared to the half-life of antibodies in human, which is about 10–21 days and as much as 25 [121]. However, the half-life estimates can vary depending on isotype and features of the immunoglobin variable region [122]. Oguit et al. showed that antibodies in infants generated from six different antigens used to immunize mothers had a decay rate of about 28–36 days depending on the antigen and that the small magnitude could be an artifact between studies done in different laboratories [123]. Yet, Tam et al., showed that a significant correlation exists between the pK from nine antibodies from human data l compared to their preclinical data [124]. For RSV specific antibodies, Munoz et al. estimated that the half-life of RSV-specific maternal antibodies in human infants was closer to 40 days [62]. We estimate the half-life in AGM to be in the range of 4.4 to 62 days, with the median half-life to be approximately 15 days. For the RSV F nanoparticle vaccine, the half-life of three doses in baboons was similar [125]. Given that the peak of disease occurs 2–3 months post birth [126], we could estimate the need to boost the expecting AGMs titers by about 64 fold [i.e., 2 × 10^6^] and 4000× [i.e., 2 × 10^12^], where the half-life is once each two weeks in NHPs, to effectively protect 3 months and 6 months post birth. In our study, we are boosting animals nearly 25 fold above pre-existing antibodies; however, one animal was an outlier with a higher (62-fold) starting antibody level. Excluding that one animal, comparing the boost response to the animals in the unvaccinated cohort has an increase of approximately 300 fold. Direct infection at 3–4 and 6 months could be the logical next step with an optimal vaccine candidate, to determine if the waning antibodies are less protective than the level closer to birth.

Together, these data suggest that African green monkeys, specifically, can serve as a reliable model to evaluate MI for candidate RSV vaccines. Seropositive pregnant AGMs when boosted with a candidate vaccine resulted in boosted antibody response observed one-month post immunization and there was noticeable transfer to offspring. Furthermore, it would be reasonable to consider an experimental challenge on the infants at various time points post birth to evaluate the efficacy and duration of the Nabs that are passively acquired from immunized mothers, which is the next logical study for this model using a vaccine candidate in development. We know that once new antigen designs, such as the mutation stabilized pre-fusion F, and adjuvant systems are identified, it is conceivable that increases in duration and potency are likely to improve [32] resulting in testing vaccines for ability to reduce viral in the respiratory tract. Evaluation of candidate vaccines for RSV administered to women during pregnancy can be de-risked utilizing the model system. PKPD relationships can be made with this model and dosing and durability relationships can be made to support exposure-response relationships typically applied in drug development and add tremendous value prior to assessment in the clinic.

## 4. Materials and Methods

### 4.1. Animal Experiments

All experiments involving laboratory animals were approved by the Institutional Animal Care and Use Committees at the University of Louisiana at Lafayette, The Mannheimer Foundation and Merck & Co., Inc., Kenilworth, NJ, USA and conducted in accordance with the Guide for the Care and Use of Laboratory Animals. Additionally, the studies adhered to the ARRIVE guidelines of the National Centre for the Replacement, Refinement & Reduction of Animals in Research (The ARRIVE guidelines 2.0: updated guidelines for reporting animal research. Originally published in PLOS Biology, July 2020. https://arriveguidelines.org/arrive-guidelines (accessed on 2 November 2021)).

### 4.2. Serology, Immunizations, and Viral Challenge

Hamadryas baboons (*Papio hamadryas*), African Green monkeys (*Chlorocebus sabaeus*) and Rhesus Macaques (*Macaca mulatta*) were domestically bred, raised, and maintained at either at the Mannheimer Foundation, Homestead, Florida or the New Iberia Research Centre (NIRC) of University of Louisiana at Lafayette, New Iberia, LA, USA. Healthy monkeys or baboons were venously bled, serum was processed and stored until evaluated. Stabilized prefusion, RSV F protein (DSCav1) [127], formulated with an aluminium phosphate adjuvant (Adju-Phos^®^ Adjuvant, InvivoGen, San Diego, CA, USA) in 0.1 mL volume was administered, intramuscularly in pregnant adult AGM, sedated with 10 mg/kg Ketamine. Approximately one-month post immunization, all monkeys were anesthetized, and a blood sample was collected. For animals that were challenged, briefly RSV seronegative animals (N = 3 each species) were anesthetized with Telazol (4–6 mg/kg) and challenged with 2 × 10^5.5^ plaque forming unit (pfu) of RSV A2 strain. The challenge virus was administered by intranasal and intratracheal inoculation, 1 mL by each route. To collect BAL samples, animals were sedated with Telazol (4–6 mg/kg) and supplementation of Ketamine (5 mg/kg) if necessary. Approximately 5 mL Hanks Balanced Salt solution (Lonza #BE10-508F) was infused directly into the lung and aspirated via a sterile French catheter and syringe. Recovered samples were supplemented with 0.1 volume of 10 × SPG and 0.1 volume of 1% gelatin, aliquoted, flash frozen and stored at −70 °C as previously described [128,129,130].

Animals were observed twice daily throughout the study for any abnormal clinical signs, signs of illness or distress. All animals were returned to their respective colonies after breaking biocontainment. All animals were socially housed prior to the start of the study. Animals were pair housed before RSV challenge and singly housed upon RSV challenge. The dimensions of the cage for singly housing were 4.3 (floor area/animal, foot square) and 30 (height, inch). For paired housing, two of these cages were placed side by side so both animals have access to both cages. Each animal’s immediate holding cage was cleaned daily. Animals were provided with object(s) to manipulate or explore. Harlan Teklad Monkey Chow, or its equivalent, was provided daily in amounts appropriate for the size of the animal. The basic diet was supplemented with fruit and novel treats including small quantities of fresh fruits, nuts, or seeds, 2 to 3 times weekly as part of the site’s environmental enrichment program. Tap water was provided ad libitum via automatic watering device. No contaminants are known to have been present in the food or water which would interfere with the results of this study. Food was withheld at least 2–3 h on days of study procedures to ensure safe sedation and was offered upon recovery from sedation.

### 4.3. Detection of Sera IgG (H+L), IgG (Fc), and IgM, Antibody Titers by ELISA

RSV specific IgG and IgM antibody were measured via ELISA in both dam and infant serum. Basically, microtiter Nunc plates were coated with RSV post fusion (F) protein at 2 μg/mL in 50 μL PBS at 4 °C overnight. The following day, plates were warmed to 16–24 °C and washed six times with PBS 0.05% Tween, followed by an hour incubation with blocking buffer consisting of 3% non-fat dry milk dissolved in PBS 0.05% Tween (PBST). After incubation and six washes with PBST, sera were diluted in blocking buffer starting at 1:50 with four-fold serial dilutions in blocking buffer. After sera incubated for two hours, plates were washed six times with PBST. 50 μL of anti-human IgG (H+L) HRP-conjugated secondary antibody was added at a 1:2000 dilution (Note: IgG (Fc) and IgM diluted at 1:5000). After one-hour incubation, plates were washed six times with PBST. 100 μL of TMB substrate was added to each well, followed by a stop solution six minutes later. Plates were read at 450 nm. Lower limit for antibody detection was the average optical density reading for blocking buffer multiplied by two. No OD value below 0.10 was included in the analysis. IgG (H+L) measured against RSV prefusion/post fusion protein. IgG (Fc) and IgM measured against RSV F prefusion protein.

### 4.4. Determination of RSV Sera Neutralizing Titers (Microneutralization Assay)

Heat-inactivated sera samples from dam and infant collected at birth and monthly through six months after birth were added in duplicate and diluted 1:4 in virus diluent (2% FBS in EMEM) followed by 2-fold serial dilutions. RSV Long virus diluted to 2000 pfu/mL in virus diluent and added to all wells (50 μL) except the cell-only control. Virus and sera were incubated for one hour at 37 °C/5% CO_2_ on a rocker. HEp-2 cells with 90–100% confluence was washed and centrifuged three times. Cells were diluted in virus diluent to reach a final concentration of 1.5 × 10^5^ cells/mL. HEp-2 cells were added to all wells (100 μL) and mixed with sera and virus. Plates were incubated for 72 h at 37 °C 5% CO_2_. Following incubation, media was removed, and plates were manually washed once with PBS. Cold 80% acetone in PBS was used to fix cells to the plate. After air-drying, plates were washed six times with PBS 0.05% Tween. Purified mouse monoclonal IgG (RSV anti-F) was diluted in assay diluent (1% BSA-PBS-0.1% Tween) at a concentration of 1.25 μg/mL and incubated for one hour. Plates were washed six times with PBST and biotinylated horse anti-mouse IgG (H+L) diluted 1:200 in assay diluent was added to each well. After one-hour incubation, plates were washed 6 times with PBST. A cocktail of IRDye 800CW Streptavidin (1:1000), Sapphire 700 (1:1000), and 5 mM DRAQ5 (1:10,000) was created and diluted in assay diluent. The mixture was added to every well and incubated for one hour in the dark. Plates were washed six times with PBST and air-dried in the dark for 20 min prior to being read on the Li-Cor Aeries Imager and an 800/700 ratio was determined. We determined the 50% endpoint titer by the following equation: (Virus Control-Cell Control)/2 + Cell Control. Serologic infection was defined as a 4-fold increase in antibody titer.

## Figures and Tables

**Table 1:** Twelve pregnant AGM were enrolled into a study. Five random female monkeys were immunized with RSV F protein with Adju-Phos® Adjuvant prior to birth. The dam was bled prior immunization. Both dam and offspring were bled on the day of birth, and serum samples were tested for anti F antibodies. The seven animals not vaccinated had lower antibody levels which was similar to the infant and was relatively low. In contrast, three boosted mothers transferred a high level of antibody to the new-born after giving birth 2, 3- or 4-weeks post vaccination. Geometric mean anti-RSV F titers of vaccinated and un-vaccinated animals are shown below. Baseline is the serum IgG determination prior the immunization of Pre F RSV protein+ Adju-Phos® or no vaccine. One month old is serum IgG determination approximately 28 days following birth. Days Prior Birth is the number of days the mother was immunized prior birth. The mother that was vaccinated and gave birth two days post vaccination was excluded from this comparison. The dam titer was 3200 at Day 0 and 204,800 at Day 28 post immunization and infant was 3200 at birth and thus shows the temporal importance of maternal antibody transfer. Antibody levels comparing vaccinated and unvaccinated mothers were significantly boosted (*p*-value < 0.001; *F*-value 36.64, Degrees of Freedom 1). Antibody levels between new-born from vaccinated versus unvaccinated mothers were significantly higher from vaccinated mothers at birth (*p*-value < 0.001; *F*-value 28.22, Degrees of Freedom 1) and one month later (*p*-value 0.001; *F*-value 21.50, Degrees of Freedom 1).

Dam	Vaccine	Baseline	Day 28 Post Vax	Infant	Birth	1 Month old	Days Prior Birth
A8M011	None	2 × 10^2^	2 × 10^2^	A18M070	2 × 10^2^	5 × 10^1^	No Vaccine
1932	None	8 × 10^2^	8 × 10^2^	A18U011	8 × 10^2^	2 × 10^2^	No Vaccine
A13M046	None	8 × 10^2^	2 × 10^2^	A18M057	2 × 10^2^	2 × 10^2^	No Vaccine
99M007	None	8 × 10^2^	2 × 10^2^	A18M067	2 × 10^2^	5 × 10^1^	No Vaccine
1799	None	3 × 10^1^	3 × 10^1^	A18U009	3 × 10^1^	3 × 10^1^	No Vaccine
A9M003	None	8 × 10^2^	2 × 10^2^	A18M073	2 × 10^2^	5 × 10^1^	No Vaccine
A7M063	None	8 × 10^2^	8 × 10^2^	A18M076	8 × 10^2^	8 × 10^2^	No Vaccine
A7M058	PreF	8 × 10^2^	5 × 10^4^	A18M071	5 × 10^4^	1 × 10^4^	14
A8M026	PreF	8 × 10^2^	5 × 10^4^	A18M075	1 × 10^4^	3 × 10^3^	21
1941	PreF	5 × 10^4^	2 × 10^5^	A18U010	2 × 10^5^	5 × 10^4^	28
A6S002	PreF	1 × 10^4^	2 × 10^5^	A18M068	3 × 10^3^	8 × 10^2^	2 days

## Data Availability

The data supporting reported results can be found in electronic notebooks stored with Merck & Co. Inc., Kenilworth, NJ, USA. Raw data that support the findings of this study are available on request from the corresponding author.
